# The inhibition of EZH2 ameliorates osteoarthritis development through the Wnt/β-catenin pathway

**DOI:** 10.1038/srep29176

**Published:** 2016-08-19

**Authors:** Linwei Chen, Yaosen Wu, Yan Wu, Ye Wang, Liaojun Sun, Fangcai Li

**Affiliations:** 1 Department of orthopedics, Second affiliated hospital, Zhejiang University School of Medicine, Hangzhou, Zhejiang Province, China; 2 Department of orthopedics, Second affiliated hospital, Wenzhou Medical university, Wenzhou, Zhejiang Province, China; 3 Department of pediatrics, Second affiliated hospital, Zhejiang University School of Medicine, Hangzhou, Zhejiang Province, China

## Abstract

The purpose of our study was to elucidate the role of the histone methyltransferase enhancer of zeste homologue 2 (EZH2) in the pathophysiology of osteoarthritis (OA) and to develop a strategy to modulate EZH2 activity for OA treatment. The expression of EZH2 in normal and OA human cartilage was compared by western blotting. The effect of EZH2 overexpression and inhibition on chondrocyte hypertrophy related gene expression was examined by real-time PCR, and histone methylation on the promoter of the Wnt inhibitor SFRP1 was analyzed using a chromatin immunoprecipitation (ChIP) PCR. Histological assessment of OA mice joint was carried out to assess the *in vivo* effects of EZH2 inhibitor EPZ005687. We found EZH2 level was significantly increased in the chondrocytes of OA patients compared to normal humans. Overexpression of EZH2 promoted Indian Hedgehog, MMP-13, ADAMTS-5 and COLX expression, while inhibition of EZH2 reversed this trend. Furthermore, the induction of EZH2 led to β-catenin signaling activation by increasing H3K27me3 on the promoter of SFRP1, while the inhibition of EZH2 silenced β-catenin signaling. Finally, intraarticular injection of EPZ005687 delayed OA development in mice. These results implicated EZH2 activity in OA development. Pharmacological inhibition of EZH2 may be an effective therapeutic approach for osteoarthritis.

Osteoarthritis (OA) is the most prevalent form of arthritis worldwide and is becoming a major public health problem^1^,^2^. Different mechanisms are involved in cartilage degradation, including inflammation, chondrocyte hypertrophic maturation, and extracellular matrix (ECM) degradation^3^,^4^,^5^,^6^. However, the underlying molecular mechanisms are not completely clarified yet.

Recently, epigenetics has been described as an important mechanism for the pathologic development of OA^7^,^8^,^9^,^10^. A significant increase in H3K9 and H3K27 trimethylation was observed at the SOX-9 promoter in OA chondrocytes^11^. The lysine-specific demethylase histone demethylase 1 (LSD1), was elevated in OA compared to normal cartilage^12^. However, to the best of our knowledge, no study has identified the role of histone methylation in OA development to date.

The polycomb repressive complex 2 (PRC2) is an essential chromatin modifier that is responsible for the transcriptional silencing of genes involved in differentiation^13^. EZH2 is the catalytic subunit of the PRC2 complex, and its C-terminal SET domain exhibits methyltransferase activity^14^. EZH2 inhibits target gene expression through the methylation of lysine 27 on histone 3 (primarily H3K27me2 and H3K27me3)^15^. For example, EZH2 reduces the expression of the Wnt antagonist secreted frizzled-related protein 1 (SFRP1) which in turn activates Wnt/β-catenin signaling^16^.

The Wnt/β-catenin signaling pathway is responsible for the activation of collagen X (COLX) and a disintegrin and metalloproteinase with thrombospondin motifs 5 (ADAMTS-5), which leads to the degradation of cartilage-specific ECM and chondrocyte hypertrophy^17^,^18^. In contrast, the expression of type II collagen (COLII) and proteoglycans (primarily aggrecan), which is controlled by SOX9, is attenuated^19^. Therefore, the balance between anabolic and catabolic factors is dysregulated in OA.

Because activation of Wnt/β-catenin signaling in articular chondrocytes leads to chondrocyte hypertrophy and ECM degradation, regulation of Wnt/β-catenin signaling by histone methyltransferase might ameliorate the development of OA. The purpose of this study was to address whether EZH2 could induce Wnt/β-catenin signaling hyperactivation via H3K27 methylation of SFRP1 in normal chondrocytes. Conversely, silencing of EZH2 could inhibit β-catenin signaling hyperactivation in OA chondrocytes. We propose that the inhibition of EZH2 activity may represent a promising strategy for OA treatment.

## Material and Methods

### Reagents and antibodies

Dulbecco’s Modified Eagle’s Medium: Nutrient Mixture F-12 (DMEM/F-12) Media was obtained from Hyclone (Utah, USA). Recombinant human and mouse IL-1β were obtained from R&D Systems (Minneapolis, MN, USA). Penicillin, streptomycin and fetal bovine serum (FBS) were obtained from Gibco BRL (Grand Island, NY, USA). EPZ005687 was obtained from MedChemExpress _(1396772-26-1). The following antibodies were used in this study: anti-EZH2 from Abcam (Cell Signaling Technology); anti-SOX9 and anti-β-catenin from Cell Signaling Technology (Danvers, MA, USA); anti-H3K27me3 (Millipore, CA, US); normal rabbit IgG (Santa Cruz, Heidelberg, Germany); anti-SFRP-1 (Abcam, Cambridge, UK); Alexa-Fluor-488- and Alexa-Fluor-545- conjugated secondary antibodies from Molecular Probes (Eugene, OR, USA); and goat anti-rabbit IRDye 800CW and goat anti-mouse IRDye 680 secondary antibodies from LI-COR Biosciences (Lincoln, NE, USA).

### Mice, human articular cartilage, and chondrocyte culture

C57BL/6 mice (Animal Center of Zhejiang University) were used in this study. Immature mice (10 days) were used to isolate knee articular chondrocytes as previously described^20^.

The normal human articular cartilage from 10 donors was obtained from femoral condyles and tibial plateaus during amputation surgery or strauma surgeries. OA human articular cartilage was obtained from 8 patients (OA grade III–IV) undergoing total knee arthroplasty. Informed consent for further use of their specimen was collected before surgery. Work with patient tissue was approved by the local ethics committee. Cartilage slices were harvested from fresh human samples and digested with 0.25% trypsin for 30 min, followed by digestion with 2 mg/ml collagenase II in DMEM/F12 with antibiotics for 6 h at 37 °C. Thereafter, cells were suspended and seeded into tissue culture flasks. Chondrocytes were grown in DMEM/F12 supplemented with 10% FBS in an atmosphere of 5% CO_2_ at 37 °C. Chondrocytes no later than the first passage were used for all experiments.

### Immunofluorescence (IF)

Chondrocytes or slides of tissue sections were fixed in 4% formaldehyde. After washing three times in PBS, they were incubated in 10% FCS for 30 min to block nonspecific sites of antibody adsorption. Then, the tissue sections or chondrocytes were incubated with appropriate primary antibodies overnight followed by secondary antibodies for 90 min. Images were captured on a Zeiss LSM510 Meta laser-scanning confocal microscope (Carl Zeiss, Thornwood, NY, USA).

### Western blotting analysis

Proteins lysed with sample buffer were loaded onto a 10% sodium dodecyl sulfate (SDS) polyacrylamide gel and blotted onto a PVDF membrane. Then the membrane was blocked in 5% BSA and incubated with the corresponding primary and secondary antibodies. After washing, the specific bands were analyzed using an Odyssey infrared imaging system (LI-COR Biosciences). Protein bands were visualized by using LiDE 100 scanner (Canon, Japan) and quantified by densitometry analysis using image J software (College of Life Science, Zhejiang university). Western blot experiments were repeated three times to confirm the results.

### Quantitative RT-PCR

Total RNA was isolated using TRIzol (Invitrogen) according to the manufacturer’s instructions. cDNA was synthesized from 1 μg of RNA with the One Step RT-PCR Kit (TaKaRa). Quantitative real-time PCR were performed using the iQTM SYBR Green Supermix PCR kit with the iCycler apparatus system (Bio-Rad). The primer sequences were as follows: for EZH2, Forward 5′-AGTGTTACCAGCATTTGGAGGG-3′, Reverse 5′-CGGTGAGAGCAGCAGCAAAC-3′; for SFRP1, Forward 5′-TGAGGCCATCATTGAACATC-3′, Reverse 5′-TCATCCTCAGTGCAAACTCG-3′; for COLX, Forward 5′-GTGTTTTACGCTGAACGATACCAA-3′, Reverse 5′-ACCTGGTTTCCCTACAGCTGATG-3′; for Indian Hedgehog, Forward 5′-CATGACCCAGCGCTGCAAGG-3′, Reverse 5′-CCTGGAAAGCTCTCAGCCGG-3′; for MMP13, Forward 5′-GTTTTG TGCTTCCTGATGATGATGTGC-3′, Reverse 5′-CGTTTTGGGATGTTTTGGGTTGGG-3′; and for GAPDH, Forward 5′-TCCCTCAAGATTGTCAGCAA-3′, Reverse 5′-GATCCACAACGGATACATT-3′. GAPDH was used as the invariant housekeeping gene internal control.

### Lentivirus transfection

Lenti-EZH2-control, Lenti-EZH2 and Lenti-shEZH2 lentiviral particles were produced by triple transfections of 293T cells (Invitrogen, Carlsbad, USA) with the vectors of pLVX-EZH2-control, pLVX-EZH2, or pLVX-shEZH2, respectively.

Chondrocytes were transfected with the lentivirus and siRNA when the cells were 30–50% confluent at a multiplicity of infection of 200; after 12 h, more than 95% of the cells were still viable, and the culture medium was changed. After 72 h, the transfected cells were harvested and gene overexpression or knockdown was verified by Western blotting.

### Chromatin immunoprecipitation analysis

Chondrocytes were fixed with 1% formaldehyde and lysed. Chromatin was sheared by sonication and pretreated with normal rabbit serum and protein A beads (Upstate/Millipore, Zug, Switzerland). For ChIP qPCR, 1–2 μg of antibody [anti-H3K27me3 or normal IgG] was added. Chromatin was precipitated with protein A beads, washed, eluted, reverse-crosslinked, digested with proteinase K and analyzed by quantitative real-time PCR.

### Animal OA model

Fifteen 4 month old male mice were divided into 3 groups and anesthetized by intraperitoneal injection of chloral hydrate (250 mg/kg). Thereafter, the OA + EPZ005687 and OA + NS groups were subjected to anterior cruciate ligment transection to induce surgical OA. The sham group was subjected to a sham operation using the same approach. Intra-articular injection of EPZ005687 (5.6 μmol/L, 50 μL) was performed through a trans-patellar tendon approach at 7, 15 and 30 days post OA surgery^21^. The control and sham groups received normal saline (NS) while the experimental group was injected with EPZ005687. The mice were sacrificed 8 weeks post-OA surgery from each group; the knee joints were dissected and processed for histological evaluation.

### Histological assessment

Knee joints were fixed in 4% paraformaldehyde, and decalcified in neutral 10% EDTA solution for 1 month at room temperature. Thereafter, the knees were dehydrated and embedded in paraffin blocks. Sections with 8 mm thickness were prepared using a microtome. Six representative sections of each joint were stained with hematoxylin-eosin (HE staining) and Safranin-Orange (SO staining)^22^. The sections closest to the maximum diameter in the sagittal position of the knee were chosen. The articular cartilage was assessed blindly by three observers using the Osteoarthritis Research Society International (OARSI) histological scoring system^23^.

### Statistical analysis

Statistical analysis was performed using the Stata 10.0 software (StataCorp LP, College Station, TX, USA). Data are expressed as the mean ± standard deviation (SD). Statistical comparisons were performed with the 2-tailed Student’s t test. Differences were considered significant when P < 0.05.

### Ethics Statement

This study was performed in strict accordance with the recommendations in the Guide for the Care and Use of Laboratory Animals of the National Institutes of Health. The protocol was approved by the Animal Care and Use Committee of the Medical Faculty of Zhejiang University (Permit Number: XI104079). All surgery was performed under chloral hydrate anesthesia, and all efforts were made to minimize suffering. The human tissue collection was approved by the Ethics Committee of Second Affiliated Hospital, Zhejiang University School of Medicine (Permit Number: 12377). Informed consent was obtained from all participants. All human tissue experiments were conducted in accordance with the approved guidelines.

## Results

### Aberrant activation of *EZH2* in human OA cartilage

**Figure 1:**
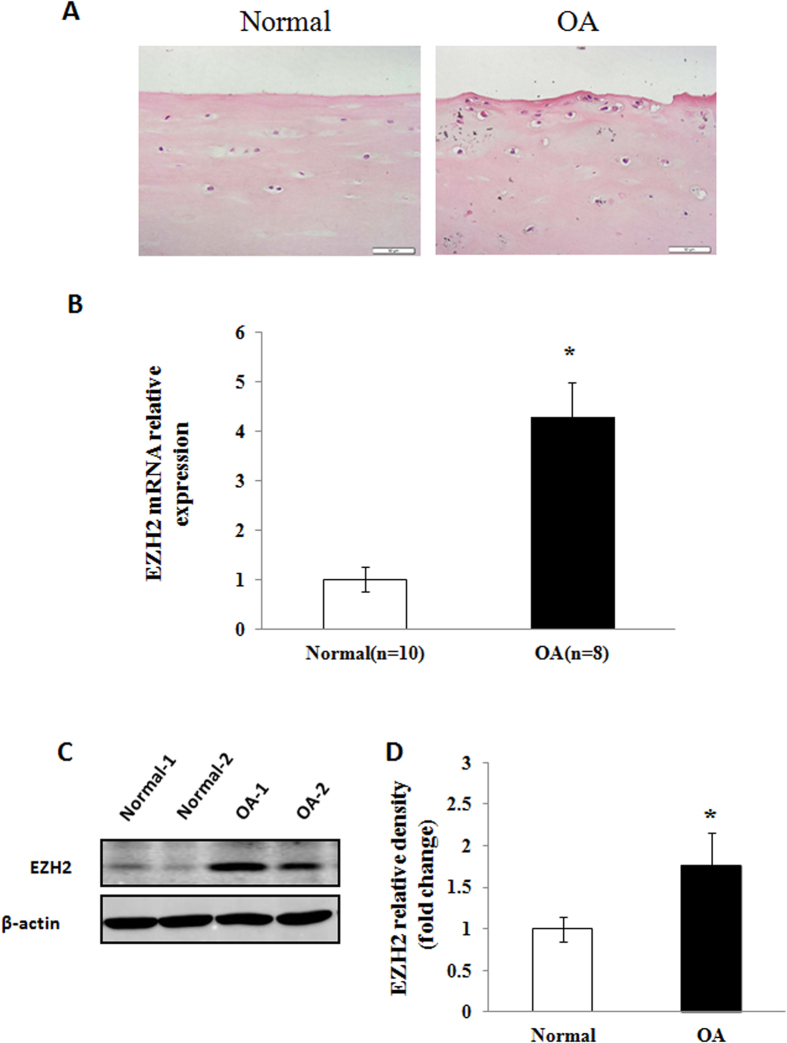
EZH2 expression in osteoarthritis (OA) and normal articular chondrocytes. (**A**) HE staining showed smooth surfaces in human articular cartilage and rough surfaces in OA human articular cartilage. Scale bars, 50 μm. (**B**) Real-time PCR analysis revealed higher expression of EZH2 mRNA in OA chondrocytes (n = 8) cultivated from OA cartilage sample compared to normal chondrocytes (n = 10). *p < 0.05. (**C**) A representative comparison of the EZH2 level of human normal and OA chondrocytes by western blot. (**D**) Quantitative analysis of western blot confirmed a significant upregulation of EZH2 expression in OA chondrocytes compared with normal chondrocytes. Values are means ± SD, *p < 0.05.

To evaluate if there is aberrabt activation of EZH2 in the development of OA, we compared the level of EZH2 in normal and OA human articular chondrocytes by Realtime PCR and Western blotting analysis. HE staining showed smooth surfaces in human articular cartilage and rough surfaces in OA human articular cartilage (Fig. 1A). Furthermore, Real-time PCR analysis revealed higher level of EZH2 mRNA in OA chondrocytes compared to normal chondrocytes (Fig. 1B). Similar results were also observed at the protein level, with increased level of EZH2 detected in the articular chondrocytes of OA patients by Western blotting (Fig. 1C). Quantification of western blot by densitometry analysis confirmed a significant upregulation of EZH2 level in OA chondrocytes compared with normal chondrocytes (Fig. 1D).

### *IL-1β* enhances the level of *EZH2* in normal chondrocytes

**Figure 2:**
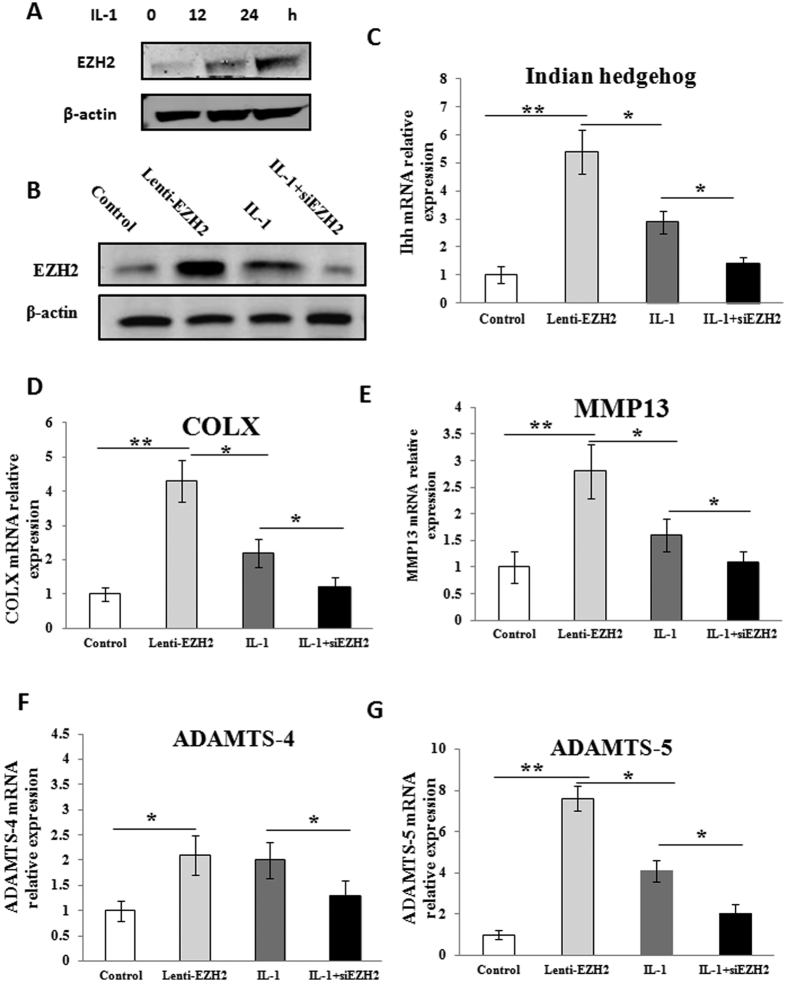
Modulation of EZH2 expression controlled hypertrophy and matrix degradation-related gene expression. (**A**) EZH2 expression increased gradually in normal chondrocytes (P2) after being treated with IL-1β (10 ng/ml) for 0, 12 and 24 hours demonstrated by Western blotting. (**B**) Successful overexpression of EZH2 in normal chondrocytes was validated by Western blotting analysis. Meanwhile, knockdown of EZH2 by siEZH2 greatly inhibited the enhanced expression of EZH2 which was induced by IL-1β treatment in normal chondrocytes. (**C–G**) Effect of overexpression and knockdown of EZH2 in normal chondrocytes on Indian Hedgehog, COLX, MMP-13, ADAMTS-4 and ADAMTS-5 expression was demonstrated by Realtime RT-PCR. Compared with control group, the expression of these catabolic genes was significantly increased by Lenti-EZH2. Similarly, IL-1β treatment for 24 h also increased these catabolic genes but the effect was weaker than Lenti-EZH2. Finally, knockdown of EZH2 greatly reduced the IL-1β-induced increase of these catabolic genes. Values are means ± SD (n = 3). *p < 0.05, **p < 0.01.

The pro-inflammatory cytokine IL-1β has been implicated as a major causative factor for OA^24^. To address whether IL-1β might modulate the level of EZH2 in normal chondrocytes, we examined the level of EZH2 in human articular chondrocytes culture system after IL-1β treatment (10 ng/ml) for 0, 12 and 24 hours. As shown in Fig. 2A, Western blotting analysis revealed that IL-1β treatment greatly increased the level of the EZH2 protein in a time-dependent manner. This result indicated that the elevation of EZH2 activity is a characteristic event during human OA development.

### Effect of *EZH2* overexpression and knockdown on the expression of catabolic genes

In OA chondrocytes, hypertrophy or the expression of catabolic genes including COLX, Indian Hedgehog, MMP-13, ADAMT-4 and ADAMT-5 are upregulated. To investigate whether EZH2 predisposes to OA phenotype, we introduced Lenti-EZH2 or siEZH2 into normal chondrocytes, the validation of the overexpression and knockdown of EZH2 was included in Fig. 2B. Overexpression of EZH2 resulted in increased level of COLX, Indian Hedgehog, MMP-13, ADAMTS-4 and ADAMTS-5 (Fig. 2C–E). Similarly, IL-1β treatment was used to induce the OA phenotype of normal chondrocytes. Increased level of hypertrophy and matrix degradation-related genes was observed but this effect was not as strong as that of Lenti-EZH2. Conversely, knockdown of EZH2 significantly reduced the IL-1β-induced increase of COLX, Indian Hedgehog, MMP-13 ADAMTS-4 and ADAMTS-5 in normal chondrocytes (Fig. 2C–E).

### Overexpression of *EZH2* decreased *SOX9* production

**Figure 3:**
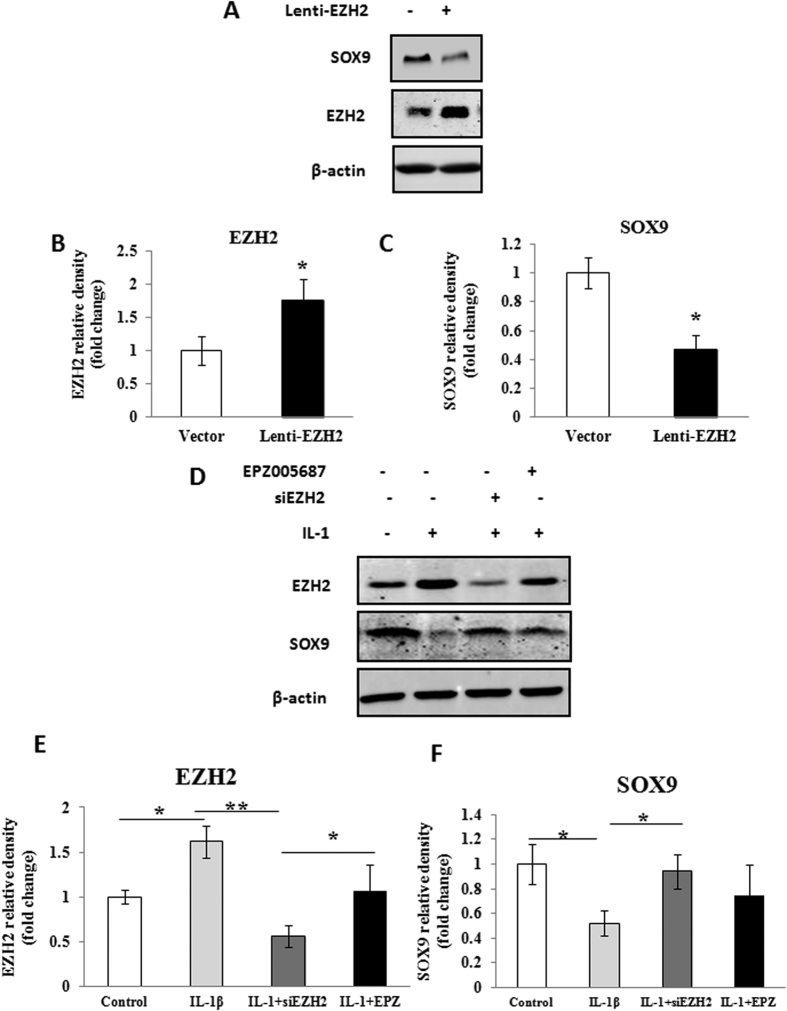
Expression of EZH2 influenced SOX9 expression in normal chondrocytes. (**A**) Normal chondrocytes (P2) were infected with Lenti-EZH2. A significant increase of EZH2 and a obvious decrease of SOX9 were demonstrated by Western-blotting. (**B,C**) A quantitative analysis of EZH2 and SOX9 protein expression confirmed the Western-blotting results. (**D**) The normal chondrocytes (P2) were treated with IL-1β (10 ng/ml) for 24 hours to induce chondrocyte pathological change followed by knockdown by siEZH2 or treatment by pharmacological inhibitor EPZ005687. The effect of each treatment was investigated by Western-blotting and quantitative analysis (**E,F**). IL-1β treatment notably enhanced EZH2 expression and inhibited SOX9 expression. Subsequently, knockdown of EZH2 by siEZH2 downregulated EZH2 expression and ameliorate the inhibition of SOX9. Meanwhile, the inhibition of SOX9 was also attenuated following treatment with the EZH2 inhibitor EPZ005687 (5.6 μmol/L). Values are means ± SD (n = 3). *p < 0.05, **p < 0.01.

Since SOX9 plays a crucial role in the regulation of COLII and aggrecan, we examined the relationship between SOX9 and EZH2. We firstly introduced Lenti-EZH2 within normal chondrocytes and measured the levels of EZH2 and SOX9. The result showed the overexpressed EZH2 significantly decreased the SOX9 level in chondrocytes (Fig. 3A). Quantification of western blot confirmed EZH2 overexpression and SOX9 inhibition (P < 0.05) (Fig. 3B,C). Moreover, IL-1β treatment induced increase of EZH2 and significant decrease of SOX9 in normal chondrocytes (Fig. 3D–F). In contrast, EZH2 expression induced by IL-1β was knocked-down by siEZH2 and resulted in recovery of SOX9 (Fig. 3D–F). Furthermore, EZH2 level induced by IL-1β was decreased following pretreatment with the EZH2 inhibitor EPZ005687 (5.6 μmol/L), with a concomitant upregulation of SOX9 (P < 0.05) (Fig. 3D–F).

### Overexpression of *EZH2* activated *Wnt/β-catenin* signaling

**Figure 4:**
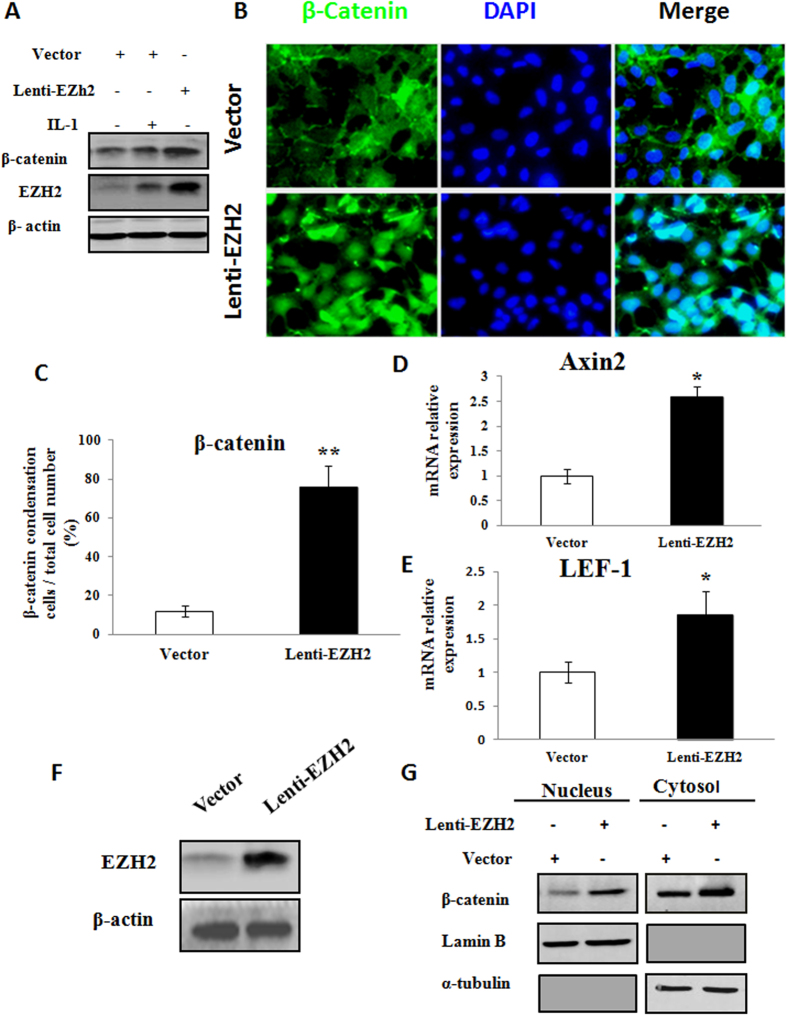
EZH2 induced pathologic changes in chondrocytes partially through regulation ofWnt/β-catenin signaling pathway. (**A**) Treatment with IL-1β (10 ng/ml) for 24 hours induced EZH2 expression and increased β-catenin expression in normal chondrocytes (P2). Meanwhile, overexpression of EZH2 by Lenti-EZH2 induced much higher increase of β-catenin expression in normal chondrocytes than IL-1β treatment. (**B**) Immunofluorescence of EZH2 revealed that β-catenin was enriched in the nuclei of normal chondrocytes transfected with EZH2, while mainly localised in cytoplasm and on cell membrane in chondrocytes transfected with vector. (**C**) Quantitative analysis confirmed β-catenin was enriched in the nuclei of chondrocytes transfected with EZH2. (**D,E**) EZH2 overexpression resulted in Wnt downstream signalling Axin2 and LEF-1 activation. (**F**) EZH2 overexpression led to the elevation of global EZH2 expression in the chondrocytes. (**G**) Cytocol/nuclear separation was undertaken to assess the enrichment of β-catenin in the nuclei of EZH2-overexpressing chondrocytes. The upregulation level of β-catenin localized in nucleus was more obvious than that remained in cytosol after infection of Lenti-EZH2. Values are means ± SD (n = 3), *p < 0.05, **p < 0.01.

Activation of β-catenin in normal chondrocytes leads to premature chondrocyte differentiation and an OA-like phenotype characterized by the upregulation of the hypertrophy-related genes^17^,^18^. As shown in Fig. 4A, β-catenin could be activated by pro-inflammatory factor IL-1β in human articular chondrocytes. More obviously, β-catenin level was significantly increased by Lenti-EZH2.

To verify the nuclear accumulation of β-catenin after EZH2 overexpression, we observed the immunofluorescence of EZH2 in normal chondrocytes. As shown in Fig. 4B, β-catenin nuclear accumulation was greatly enhanced in normal chondrocytes transfected with EZH2. Quantitative analysis confirmed nuclear condensation of β-catenin in chondrocytes transfected with EZH2 was greater than that in chondrocytes transfected with vector (Fig. 4C). Furthermore, EZH2 overexpression resulted in the upregulation of the downstream factors Axin2 and LEF-1 (Fig. 4D,E).

To check the accumulation level of β-catenin in the nuclei, we performed cytocol/nuclear separation and confirmed that β-catenin was enriched in the nuclei in normal chondrocytes transfected with EZH2 (Fig. 4F,G). The Western blot showed increase level of β-catenin localized in nucleus was more obvious than that remained in cytosol after infection of Lenti-EZH2. This result proved that overexpression of EZH2 could promote the transportation of β-catenin from cytoplasm to nucleus.

### *SFRP-1* expression was decreased in *OA* chondrocytes

**Figure 5:**
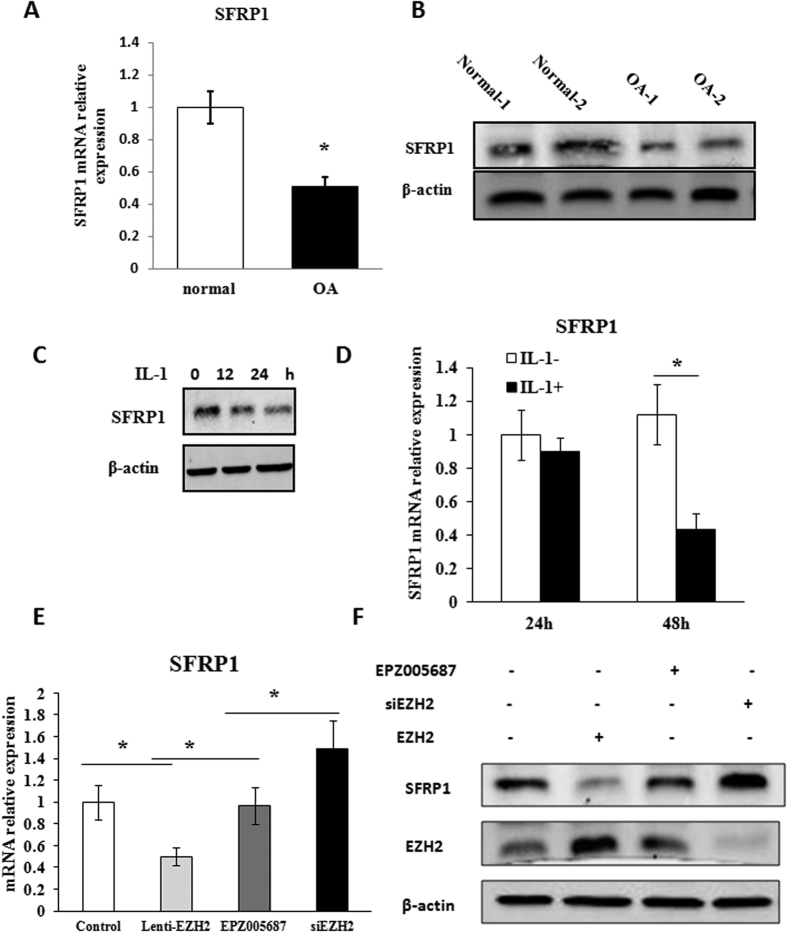
Wnt inhibitor SFRP1 was inhibited by EZH2 in OA chondrocytes. (**A,B**) The mRNA level and protein expression of SFRP1 were significantly higher in the normal chondrocytes than in OA chondrocytes demonstrated by real-time-PCR and western blot, respectively. (**C,D**) SFRP1 expression level was decreased when normal chondrocytes were treated with IL-1β as determined by real-time-PCR and Western blotting. (**E,F**) The inhibited mRNA and protein expression of SFRP1 induced by Lenti-EZH2 infection in normal chondrocytes were partly relieved by EZH2 inhibitor EPZ005687, while greatly enhanced by siEZH2 infection. Values are means ± SD (n = 3), *p < 0.05, **p < 0.01.

The expression of the Wnt inhibitor SFRP1 was dysregulated in the synovium of OA^25^. To evaluate whether SFRP1 might be silenced in the chondrocytes of OA, its differential level between OA chondrocytes and normal chondrocytes was assessed. The level of SFRP1 mRNA and protein expression was significantly reduced in OA chondrocytes compared with normal chondrocytes (Fig. 5A,B). The level of SFRP1 was also decreased in IL-1β-induced normal chondrocytes (Fig. 5C,D).

### The expression of *SFRP-1* is regulated by *EZH2*

To determine whether EZH2 might be involved in the silencing of SFRP-1 in OA chondrocytes, we investigated the regulation of SFRP-1 by siEZH2 in Lenti-EZH2 infected normal chondrocytes. As shown in Fig. 5E & FSFRP1 expression was significantly inhibited by Lenti-EZH2 infection. Thereafter, SFRP1 mRNA and protein expression levels were significantly increased after EPZ005687 and siEZH2 treatment. This result indicates that the activation of Wnt/β-catenin signaling is subjected to EZH2 mediated silencing of SFRP1.

### The binding affinity of EZH2 and trimethylation status within *SFRP-1* promoter differ between OA and normal chondrocytes

**Figure 6:**
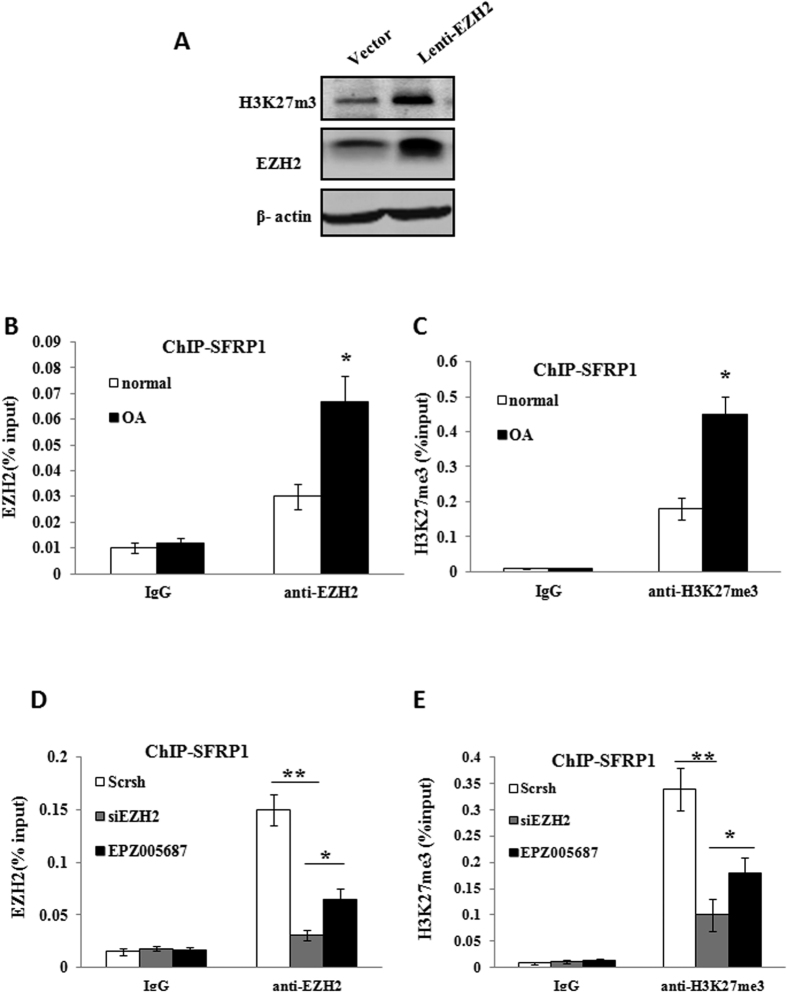
The state of H3K27 trimethylation at the promoter of SFRP1 was determined by the binding affinity of EZH2. (**A**) Western-blotting analysis revealed that the global state of H3K27 trimethylation was significantly elevated in chondrocytes infected with Lenti-EZH2 compared with control. (**B**) The promoter of SFRP1 was ChIP-ed with anti-EZH2 antibody or IgG control. OA chondrocytes showed a higher level of EZH2 occupation at the promoter of SFRP1. (**C**) The promoter of SFRP1 was ChIP-ed with anti-H3K27me3 antibody or IgG control. The repressive trimethylation of H3K27 at the SFRP1 promoter was higher in OA chondrocytes compared with normal chondrocytes. (**D**) Knockdown of EZH2 by siEZH2 or EPZ005687 reduced EZH2 binding to the promoters of SFRP1 in OA chondrocytes. (**E**) Inhibition of EZH2 by siEZH2 or EPZ005687 decreased H3K27me3 levels at the promoters of SFRP1 in OA chondrocytes. Values are means ± SD (n = 3), *p < 0.05, **p < 0.01.

The global state of H3K27 methylation was investigated to study the effect of Lenti-EZH2 on the transcriptional activity in normal chondrocytes. The result revealed that the H3K27 methylation was significantly elevated in chondrocytes infected with Lenti-EZH2 (Fig. 6A). To determine if the binding affinity of EZH2 within the SFRP-1 promoter differs between OA and normal chondrocytes, the promoters of SFRP-1 was ChIP-ed with anti-EZH2 antibody, the result showed EZH2 had a higher affinity to the SFRP-1 promoter in OA chondrocytes (Fig. 6B). In order to further address whether the presence of H3K27me3 (a silenced chromatin marker) on the SFRP-1 gene promoter differed between OA and normal chondrocytes, we performed PCR from ChIP fragments. The SFRP1 promoter was analyzed by precipitation with antibodies against H3K27me3. Compared with normal chondrocytes, there was a significant increase in repressive H3K27me3 on SFRP1 promoter areas in OA chondrocytes (Fig. 6C). This data indicate that the EZH2-mediated silencing of SFRP1 is due to increased H3K27me3 on its promoter.

### Silencing of *EZH2* decreased H3K27me3 occupation of *SFRP1* promoter areas

Because repressive H3K27 trimethylation predominated on the SFRP1 promoter in OA chondrocytes, we investigated whether siEZH2 or EPZ005687 could enhance SFRP1 activity. As shown in Fig. 6D, there was a significant decrease in EZH2 binding on SFRP1 promoter areas following siEZH2 or EPZ005687 treatment in OA chondrocytes. As a consequence, the repressive trimethylation status of H3K27 was greatly relieved by inhibition of EZH2 (Fig. 6E). These data suggest that the hyperactivation of Wnt/β-catenin signaling in OA chondrocytes might be reversed by siEZH2 *in vitro*.

### Effects of the *EZH2* inhibitor EPZ005687 on OA chondrocytes and mouse OA development

**Figure 7:**
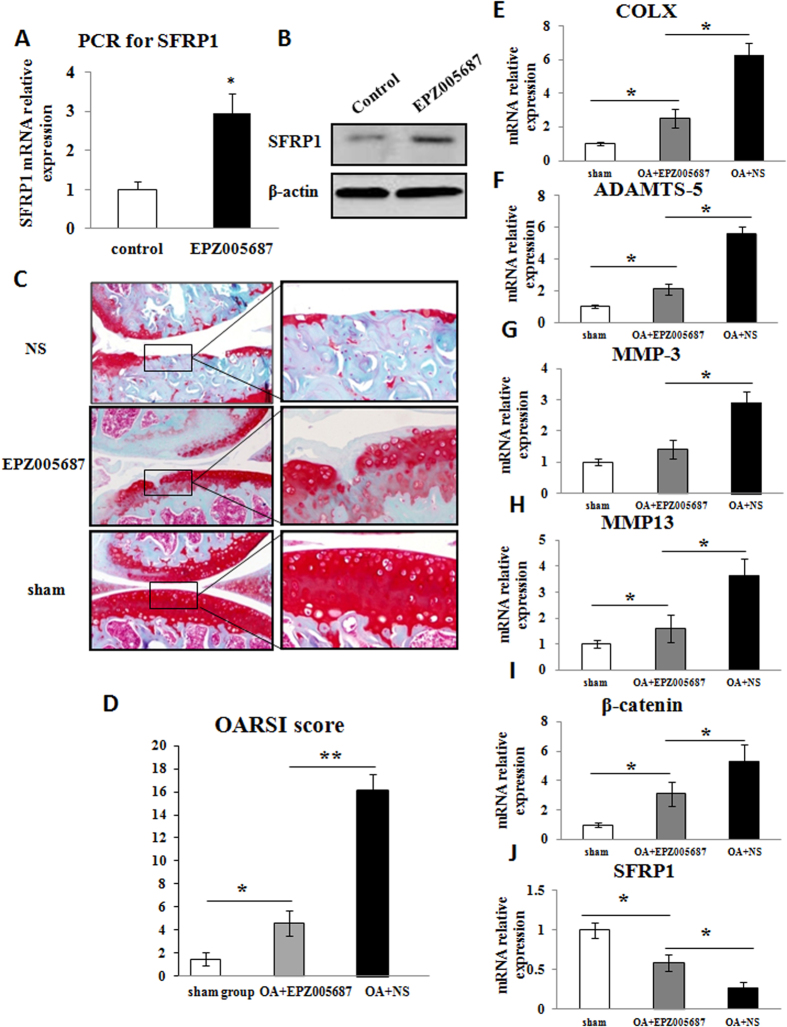
Inhibition of EZH2 activated SFRP1 in OA chondrocytes and delayed OA development *in vivo*. (**A**) EPZ005687, a strong inhibitor of EZH2, enhanced the activity of SFRP1 in human OA chondrocytes shown by Realtime PCR. (**B**) EPZ005687 revived the inhibited SFRP1 in OA chondrocytes shown by Westernblot. (**C**) EPZ005687 delayed OA development demonstrated by SO staining. Scale bars, 200 & 50 μm. (**D**) OARSI scoring of OA severity upon EPZ005687 treatment. The *in vivo* experiments clearly demonstrated the therapeutic effects of EPZ005687 on OA cartilage degeneration in mice. (**E–J**) The cartilage samples from sham, OA + EZP205687 and OA + NS group were examined for the expression of COLX, ADAMTS-5, MMP3, MMP13, SFRP1 and β-catenin by Realtime PCR. The results confirmed the protective effect of EPZ005687 on chondrocytes. Values are means ± SD (n = 3). *p < 0.05, **p < 0.01.

To explore the effect of EZH2 depletion on the protection of chondrocytes during OA development, we selected a potent and selective small-molecule inhibitor of EZH2 (EPZ005687). EPZ005687 has greater than 500-fold selectivity against 15 other protein methyltransferases. Firstly, we evaluated the augmented effect of EPZ005687 on SFRP1 expression in OA chondrocytes after *in vitro* culture. The realtime-PCR revealed that the activity of SFRP-1 in OA chondrocytes was increased following EPZ005687 treatment (Fig. 7A). Meanwhile, EPZ005687 enhanced the activation of SFRP-1 in OA chondrocytes by Western blot (Fig. 7B). The mouse OA model and sham model accepted EPZ005687 intra-articularly injection surgery. Eight weeks later, the sham-operated group showed smooth cartilage surfaces and conserved SO staining in the knee joint. The chondrocytes arrangement was also conserved, with one or two layers of tangentially arranged cells in the superficial zone and columns of round cells in the deep zones. In contrast, operated knees in the OA + NS group exhibited classical OA features evidenced by the extensive loss of cartilage and the disorganized chondrocyte arrangement. Additionally, we observed localized superficial cartilage loss, a reduction in the cartilage thickness and pale SO staining in the OA + EPZ005687 group (Fig. 7C).The OARSI scores were 1.5 ± 0.54 in the sham group, 4.6 ± 1.10 in the OA + EPZ005687 group and 16.1 ± 1.4 in the OA + NS group. The difference was significant between each group (Fig. 7D). To further confirm the *in vivo* results, we collected the cartilage samples from each groups and investigated the expression of COLX, MMP13, SFRP1, β-catenin, as well as the enzymes of extracellular matrix breakdown such as MMP3 and ADAMTS-5 by Realtime PCR. The results confirmed the protective effect of EPZ005687 on articular cartilage (Fig. 7E–J).

## Discussion

In this study, we examined the role of EZH2 in the development of OA and the underlying mechanism. We found that: (1) The level of EZH2 was increased during OA development; (2) The underlying mechanism involved the overexpression of EZH2, which promoted chondrocyte hypertrophy by activating Wnt/β-catenin signaling; and (3) Our work demonstrated a new therapeutic strategy for OA therapy through the inhibition of the aberrant activation of EZH2 with EPZ005687.

Histone modifications play critical roles in regulating both global and stage-specific gene expression. Methylation on histones H3K4, H3K36 and H3K79 is generally associated with gene activation, whereas methylation on histones H3K9 and H3K27 is generally associated with gene repression^15^. Histone lysine methylation is dynamically regulated by site-specific methyltransferases and demethylases^26^,^27^. EZH2 (the catalytic subunit of PRC2) is responsible for the methylation of H3K27 in cells. Conversely, deletion of EZH2 dramatically decreases H3K27me3 on PRC2 target genes. EZH2 has been found to play an important role in the pathogenesis and treatment of different cancers^28^,^29^. In the field of autoimmune disorder research (i.e., RA), the expression of EZH2 has been demonstrated to be hyperactivated in rheumatoid arthritis synovial fibroblasts (RASF)^30^. For the first time, our study compared the level of EZH2 between OA chondrocytes and normal chondrocytes. The results showed an elevated level of EZH2 in the articular chondrocytes of OA patients. Additionally, the hyperactivation of EZH2 could be induced by IL-1β (a well-known catabolic culprit of proteoglycan matrix loss) in a time-dependent manner^31^.

Wnt/β-catenin signaling is a powerful stimulator of chondrocyte matrix degradation^17^. Activation of Wnt signaling also decreased the proliferation and differentiation of normal mesenchymal progenitor cells (MPCs)^32^. By inducing β-catenin nuclear accumulation, EZH2 could subsequently enhance the level of COLX, Indian Hedgehog, MMP-13, ADAMTS-4 and ADAMTS-5. Our data confirmed that β-catenin might function downstream of overexpressed EZH2 in modulating pathological changes in OA chondrocytes. Our results also implied that overexpression of EZH2 inhibited SOX9 expression. The expression of ECM anabolic factors (i.e., COLII and aggrecan) was also suppressed^19^,^33^.

Hyperactivation of β-catenin in articular chondrocytes predisposes adult mice to the OA phenotype^17^,^34^,^35^. Therefore, investigating how EZH2 interacts with Wnt/β-catenin and triggers its cellular translocation is of interest. Because SFRP1 possesses a domain similar to the Wnt-receptor frizzled proteins and can inhibit Wnt receptor binding to downregulate pathway signaling^36^, reduced SFRP1 expression not only leads to the activation of Wnt/β-catenin signaling but also renders the articular cartilage prone to the development of OA^37^.

SFRP1 has already been identified as a direct target gene of EZH2 in RA. Michelle Trenkmann identified sFRP1 as a direct target gene of EZH2 in RASF^30^. In our experiment, we found EZH2 activated β-catenin signaling by suppressing SFRP1. Both studies link EZH2 to sFRP1. However, there is evidence that sFRP3 loss of function also predisposes to OA^38^,^39^. Therefore, EZH2 may activate β-catenin signaling through sFRP3 or any other WNT inhibitor. Our study does not rule out the possibilities of EZH2 in regulating any other WNT inhibitors in OA development but does highlight the important role of SFRP1 as EZH2 target in mediating wnt signalling pathway in OA.

Our data indicated that the level of SFRP1 was lower in OA chondrocytes compared with normal chondrocytes, and the epigenetic landscape of SFRP1 in the OA chondrocytes was changed towards a transcription-inhibiting state. Overexpression of EZH2 activated Wnt/β-catenin signaling by decreasing H3K27me3 on SFRP1 promoter areas. Moreover, silencing EZH2 deactivated Wnt/β-catenin signaling by decreasing H3K27me3 on SFRP1 promoter areas. Our results demonstrated that activated β-catenin signaling in OA chondrocytes could be epigenetically reversed by the removal of H3K27me3 from the SFRP1 promoter by siEZH2. Furthermore, our study provided solid evidence to confirm that the inhibition of EZH2 activity by the small molecule inhibitor EPZ005687 enhanced SFRP1 activity in OA chondrocytes and delayed OA development in mouse OA model, as evidenced by the reduced cartilage degradation, decreased chondrocyte hyperthrophic marks, enzymes of extracellular matrix breakdown, β-catenin and increased SFRP1 in OA chondrocytes, as well as decreased OARSI score.

## Conclusion

In conclusion, we demonstrated that EZH2 acts as a crucial mediator of OA cartilage destruction by upregulating the expression of catabolic genes through the Wnt/β-catenin signaling pathway. Moreover, we found that EZH2 depletion resulted in β-catenin signaling silencing by removing H3K27me3 from the SFRP1 promoter. Our results indicate that developing a specific inhibitor of EZH2 may be beneficial for the prevention or therapeutic treatment of osteoarthritis.

## Additional Information

**How to cite this article**: Chen, L. *et al*. The inhibition of EZH2 ameliorates osteoarthritis development through the Wnt/β-catenin pathway. *Sci. Rep.* **6**, 29176; doi: 10.1038/srep29176 (2016).
